# Giant electrocaloric effect in a wide temperature range in PbTiO_3_ nanoparticle with double-vortex domain structure

**DOI:** 10.1038/s41598-017-18275-0

**Published:** 2018-01-10

**Authors:** C. Ye, J. B. Wang, B. Li, X. L. Zhong

**Affiliations:** School of Materials and Engineering, Xiangtan University, Hunan Xiangtan, 411105 China

## Abstract

Electrocaloric effect (ECE) has the potential applications in solid-state refrigeration with the features of high efficiency and environmentally friendly. Large adiabatic temperature change in a wide temperature range is needed for electrocaloric effect to meet the requirement of commercially application. In this work, giant electrocaloric effect is found in PbTiO_3_ nanoparticle with double-vortex domain structure in a wide temperature range by using phase field method, which the lowest and highest adiabatic temperature change (Δ*T*) is 7.2 K and 16.5 K, respectively. The influence of misfit strain on the ECE of PbTiO_3_ nanoparticle with the double-vortex domain structure is investigated, and results show that the compress misfit strain can enhance the ECE, but the tensile misfit strain reduces the ECE. This work reveals a way to obtain giant ECE of ferroelectric materials by domain engineering and strain engineering in a wide temperature range.

## Introduction

Electrocaloric effect (ECE), a temperature change of a given material in response to external electric field, has attracted considerable attention because it can be used as solid-state refrigeration to replace Freon-based cooling systems with the feature of high efficiency^1–5^. Large ECEs near ferroelectric-paraelectirc (F-P) phase transition have been reported in the low dimensional ferroelectric materials^6–8^, which proved that low dimensional ferroelectric materials have great potential for practical applications and can be commercially exploited for electronic devices. However, the working temperature of many devices near the room temperature, which much lower than the temperature of F-P phase transition^9–11^. Therefore, it is important to find an effective way to design and develop ferroelectric materials which can generate giant adiabatic temperature change near the room temperature.

At room temperature, domains are formed in ferroelectric materials when polarization has the same direction. The domain engineering, which control and adjust the properties of ferroelectric materials through changing the domain structure and number, has been proved an effective way to control and adjust the ECE of ferroelectric materials^12–15^. For example, Peng *et al*. reported that the field-induced polar nanodomain formation and alignment could induced a twin-peak ECEs^12^. Wang *et al*. found that the transition from multidomain to monodomain driven by temperature can largely enhance the ECE of PbTiO_3_^13^. Especially, our previous works have found a large temperature change related to the toroidal moment change in the ferroelectric nanoparticle with vortex domain structure^16,17^. These works indicate that the ferroelectric nanostructure with the vortex domain structure can provide an effective way to achieving a large ECE through domain engineering.

In addition, in order to better satisfy the requirement of commercially application, it has necessary to further improve the ECE of ferroelectric nanostructure with vortex domain structure near the room temperature. It is noted that, controlling and adjusting the properties of ferroelectric materials through the strain, what is the so called strain engineering, is another effective way to control and adjust the properties of ferroelectric materials^18–20^. For example, Haeni *et al*. has observed a Curie temperature *T*_C_ shift of hundreds of degrees in strained ferroelectric thin films owing to the strong coupling between strain and ferroelectricity^19^. The epitaxial strain from the substrate could be applied to increase of *T*_C_ and raise the produce room-temperature ferroelectricity. Cruz *et al*. find that a film-substrate misfit strain may significantly affect the thermodynamic stability of domain walls in epitaxial BiFeO_3_ film^20^. So, it is an effective way to improve the ECE of ferroelectric nanostructure with vortex domain structure by combining the domain engineering and strain engineering.

In this letter, the influence of misfit strain on the ECE of PbTiO_3_ ferroelectric nanoparticle with a double-vortex domain structure is investigated by using phase field method. A large adiabatic temperature change is found in the PbTiO_3_ nanoparticle. And the mechanism of this large adiabatic temperature change is analyzed in detail.

## Results

### The ECE of PbTiO_3_ (PTO) nanoparticle with double-vortex domain structure at room temperature

**Figure 1:**
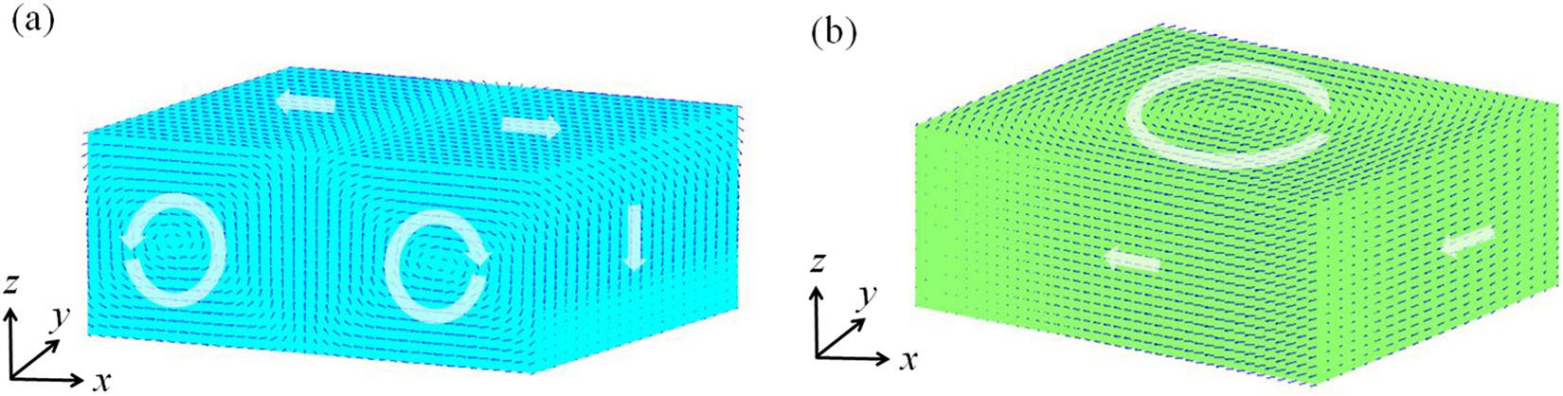
(**a**) The initial double-vortex domain structure of PbTiO_3_ nanoparticle at room temperature; (**b**) the single-vortex domain structure of PbTiO_3_ nanoparticle under the vorticity vector of circular electric field *Q* = 0.5 mV/Å^2^.

In this work, a 3D model of 40 × 20 × 20 discrete grids is employed to denote PTO nanoparticle, and grid length Δ*x* ^*^ = Δ*y* ^*^ = Δ*z* ^*^ = 1 with a cell size of Δ*x* = Δ*y* = Δ*z* = 1 nm. In order to obtain double-vortex domain structure, the polarization boundary condition of PTO nanoparticle is set as zero boundary condition and open-circuit electrical boundary condition is employed to solve Eq. (1), and time step Δ*t* ^*^ = 0.004. Due to the polarization boundary condition is zero in this paper, the surface energy is not taken into account. Figure 1(a) shows the simulation result of the initial domain structure of PTO nanoparticle at room temperature. Figure 1(a) show that there is a double-vortex domain structure which one is clockwise vortex domain and another is anticlockwise vortex domain in *x-z* plane. The formation of vortex domain is to keep the system stable through reduce the depolarization energy, and single vortex domain will turn to multi-vortex domain if the aspect ratio of nanoparticle increases^21^. In this paper, the aspect ratio of *x-z* plane is 2 which induced the formation of double-vortex domain in *x-z* plane. And the two vortex domains have opposite vortical direction can be explained as to minimize the domain wall energy^22^. In order to investigate the ECE of PTO nanoparticle with the double-vortex domain structure, a circular electric field ${{\boldsymbol{E}}}^{cir}=\frac{1}{2}{\boldsymbol{Q}}\times {\boldsymbol{r}}$ is employed^23^, where ***E*** is electric field strength, ***Q*** is the vorticity vector of circular electric field and ***r*** denotes spatial vector. Figure 1(b) shows the domain structure of PTO nanoparticle after the circular electric field was applied. The original domain structure of PTO nanoparticle changed into a new single-vortex domain structure under the circular electric filed. And the final adiabatic temperature change Δ*T* is 3.69 K under the condition *Q* = 0.5 mV/Å^2^.

**Figure 2:**
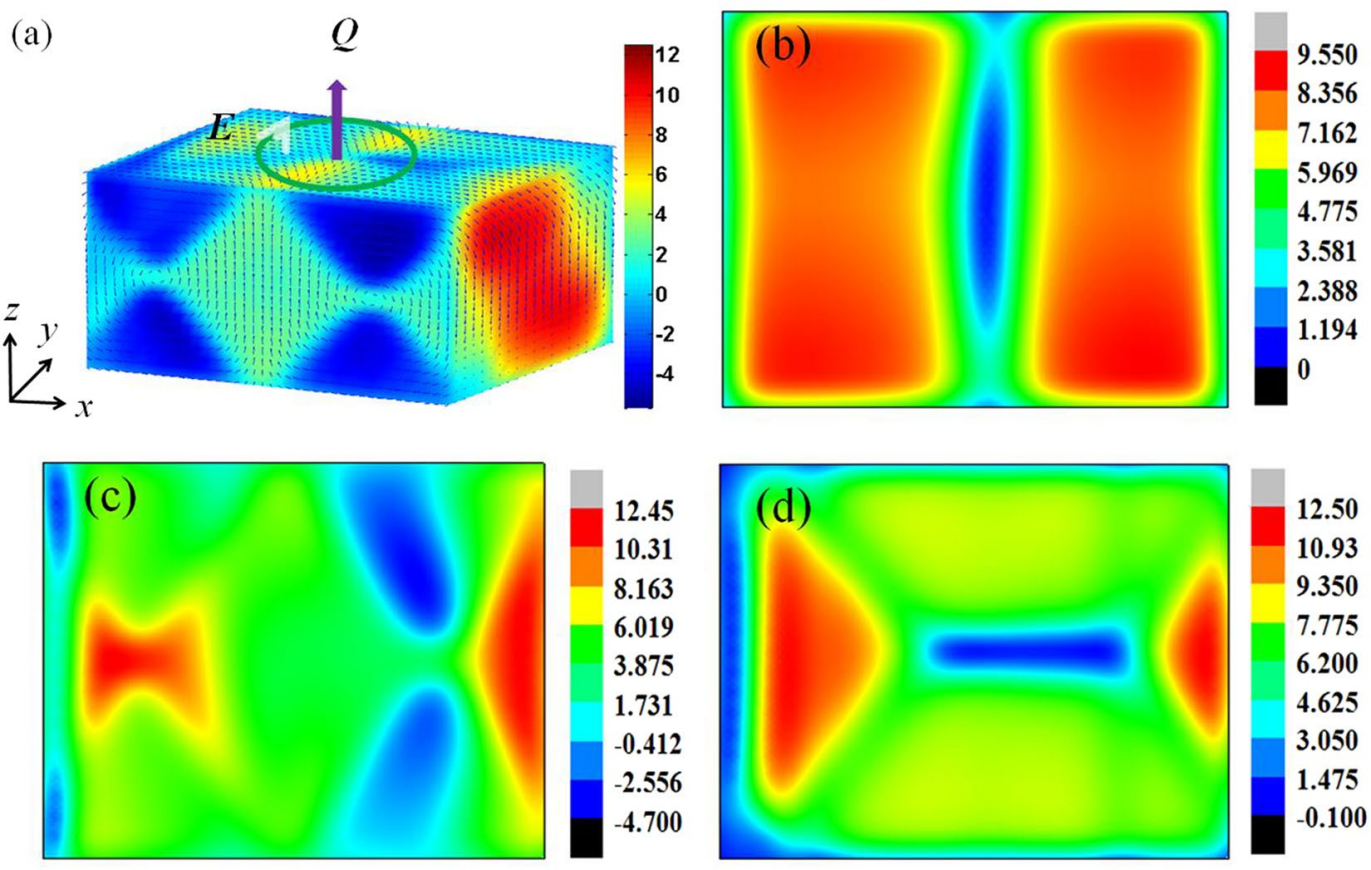
(**a**) The adiabatic temperature change of PbTiO_3_ nanoparticle with the double-vortex domain in 3D under the vorticity vector of circular electric field *Q* = 0.5 mV/Å^2^, where arrows represent the domain structure and color denotes the magnitude of adiabatic temperature change; (**b**) the *y-z* plane in the middle of PbTiO_3_ nanoparticle; (**c**) the *x-z* plane in the middle of PbTiO_3_ nanoparticle; (**d**) the *x-y* plane in the middle of PTO nanoparticle.

Figure 2(a) shows the distribution of adiabatic temperature change in 3D under the circular electric field of 0.5 mV/Å^2^. It can be seen that the temperature change of the PTO nanoparticle is different on *x-y*, *x-z*, and *y-z* surface. On *x-z* surface, the negative ECE (Δ*T* < 0) generated after the circular electric field is applied. However, most of the area generate positive ECE(Δ*T* > 0) on *x-y* surface. Apart from the variability of adiabatic temperature change along different axis, the adiabatic temperature change are also different between planes along the same axis. Figures 2(b–d) are the slices in the middle PTO nanoparticle along *x*, *y*, *z* axis, respectively. It is apparently that the distribution of adiabatic temperature change is different between the *y-z* middle plane (Fig. 2(b))and *y-z* surface. The variability of adiabatic temperature change in 3D is caused by the difference of domain structure along different axis. Despite the local temperature change is huge in some area, the final temperature change is small (Δ*T* = 3.69 K) because of the inhomogeneity of adiabatic temperature change in 3D.

### The ECE of the PTO nanoparticle with double-vortex domain structure at room temperature

**Figure 3:**
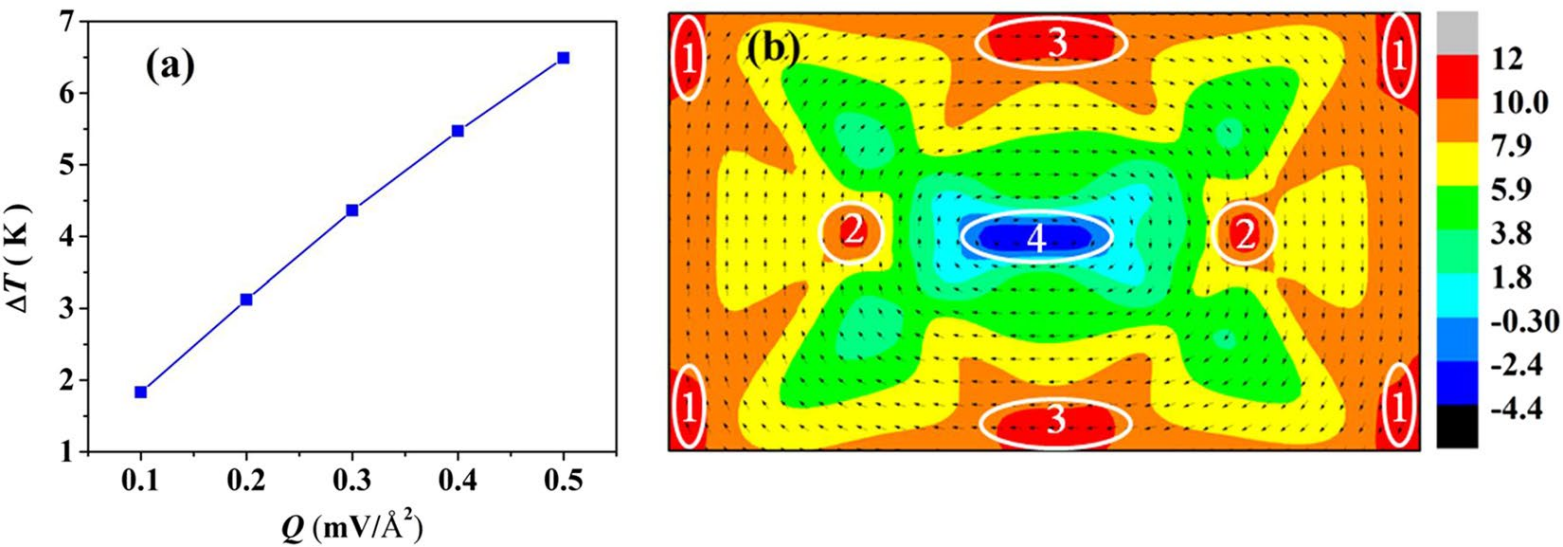
(**a**) The adiabatic temperature change of PbTiO_3_ nanoparticle with the double-vortex domain under different value of circular electric filed at room temperature; (**b**) the domain structure and the distribution of adiabatic temperature change of PbTiO_3_ nanoparticle under the vorticity vector of circular electric field *Q* = 0.5 mV/Å^2^, where arrows represent the domain structure and color denotes the magnitude of adiabatic temperature change.

The adiabatic temperature change under different value of the vorticity vector of circular electric field is exhibited in Fig. 3(a). As it shown in Fig. 3(a), the adiabatic temperature change (Δ*T*) increases from 1.8 K to 6.5 K when the vorticity vector of circular electric field (*Q*) increases from 0.1 mV/Å^2^ to 0.5 mV/Å^2^, which reveals that the ECE of PTO nanoparticle with double-vortex domain can be improved by the strength of circular electric field. A further study shows that the large adiabatic temperature change associates with the domain structure change closely under the circular electric field. The simulation result shows that double-vortex domain turns to a single-vortex domain when external circular electric filed is applied, which is shown in Fig. 1(a) and (b). Similar results are gotten when other value of circular electric field is applied. For the purpose of further investigating the relationship between ECE and domain structure change, the adiabatic temperature change distribution profile under the vorticity vector of circular electric field *Q* = 0.5 mV/Å^2^ is exhibited in Fig. 3(b). As can be seen in Fig. 3(b), large positive ECE (Δ*T* > 0) and negative ECE (Δ*T* < 0) are generated in local area, and the final adiabatic temperature change is 6.5 K under the vorticity vector of circular electric field *Q* = 0.5 mV/Å^2^ after thermal conduction.

The generation of positive and negative ECE can be explained through the domain structure change. As it has mentioned in previous paragraph, the double-vortex domain could turn into a single-vortex domain when the circular electric field is applied, the polarization of the new generated vortex center decreases largely (marked as 4 in Fig. 3(b)), so it induced the large negative ECE according to Eq. (7). On the contrary, the two vortex centers of the double-vortex domain, where are marked as 2 in Fig. 3(b), will disappear and finally form a single domain area when the single-vortex domain begin to form, so the polarization of these area increases largely and generates large positive ECE. It is the same to the areas 1 and 3 where could generate large positive ECE too. Besides, the areas of positive ECE is larger than the negative ECE. The unequal positive ECE and negative ECE are generated, and large adiabatic temperature change is obtained, finally.

### The influence of misfit strain on the ECE at room temperature

**Figure 4:**
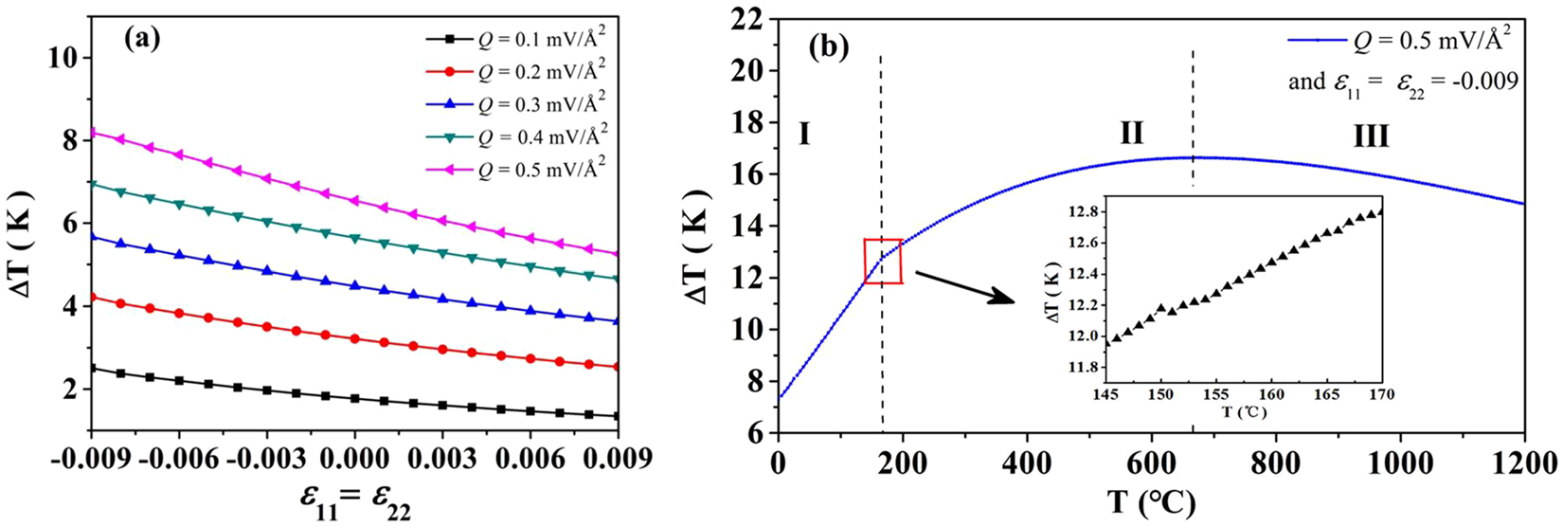
(**a**) The influence of misfit strain on the ECE of PbTiO_3_ nanoparticle with the double-vortex domain under different value of vorticity vector of circular electric field at room temperature; (**b**) the ECE of PbTiO_3_ nanoparticle with the double-vortex domain in different temperature under the vorticity vector of circular electric field *Q* = 0.5 mV/Å^2^ and equiaxial misfit strain *ε*_11_ = *ε*_22_ = −0.009.

The misfit strain induced by the lattice constant mismatch between the ferroelectrics and substrate has an important influence on the properties of ferroelectric materials^18,19^. The equiaxial misfit strain (*ε*_11_ = *ε*_22_) is employed to investigate the influence of misfit strain on the ECE of PTO nanoparticle with the double-vortex domain structure, at shown in Fig. 4(a). The influence of tensile misfit strain (*ε*_11_ = *ε*_22_ > 0) and compress misfit strain (*ε*_11_ = *ε*_22_ < 0) on ECE is different. The adiabatic temperature change increases as the absolute value of the compress misfit strain increases, which means compress can improve the ECE of PTO nanoparticle with the double-vortex domain structure. Tensile misfit strain has a opposite influence, which it reduces the ECE as the absolute value of the tensile misfit strain increases.

### The ECE of PTO nanoparticle at different temperature

**Figure 5:**
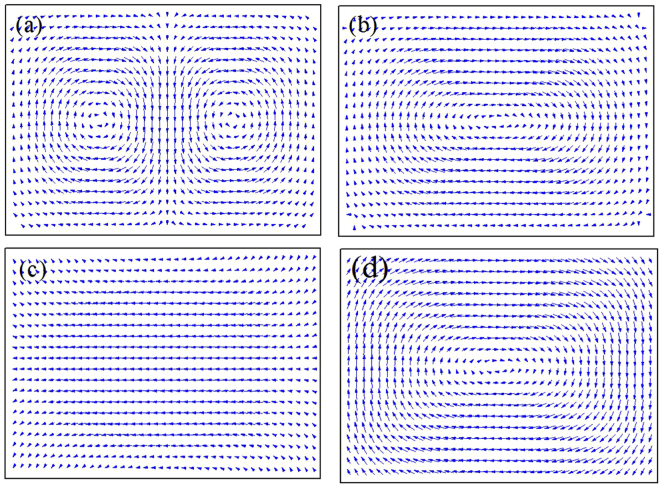
The domain structures of PbTiO_3_ nanoparticle at different temperature with the equiaxial misfit strain *ε*_11_ = *ε*_22_ = −0.009 in *x-z* plane, (**a**) 150 °C; (**b**) 151 °C; (**c**) 165 °C; (**d**)175 °C.

Apart from the giant adiabatic temperature change, a wide temperature range with large adiabatic temperature change is another important factor which limits the commercially application of ECE as the solid refrigeration. In this paper, the ECE of PTO nanoparticle with the double-vortex domain structure at different temperature is investigated. Figure 4(b) shows the adiabatic temperature change of the PTO particle at different temperature under the vorticity vector of circular electric field *Q* = 0.5 mV/Å^2^ and equiaxial misfit strain *ε*_11_ = *ε*_22_ = −0.009. Giant ECE is obtained at the temperature range 0 ~ 675 °C, and the lowest and highest adiabatic temperature change is 7.2 K and 16.5 K, respectively. The PTO nanoparticle shows different electrocaloric property in different temperature range. In the region I where the temperature range is 0 ~ 150 °C, the Δ*T* of PTO nanoparticle increases linearly as the temperature increases. When the temperature is higher than 165 °C (region II and III in Fig. 4(b)), the Δ*T* changes as a fourth order curve, and the equation of the curve can be written as ${\rm{\Delta }}T={{A}}_{1}{T}^{4}+{{A}}_{2}{T}^{3}+{{A}}_{3}{T}^{2}+{{A}}_{4}T+{B}$, where *A*_1_ = 2.77 × 10^−2^, *A*_2_ = 3.26 × 10^−5^, *A*_3_ = 1.40 × 10^−8^, *A*_4_ = 2.17 × 10^−12^, *B* = 8.96. At the temperature 675 °C, the adiabatic temperature change has the largest value (Δ*T* = 16.5 K). The difference of electrocaloric property of PTO particle in region I and II (Fig. 4(b)) attribute to the different domain transformation. Figure 5 shows the domain structure of PTO nanoparticle in different temperature. The domain structure of PTO nanoparticle is double-vortex domain as shown in Fig. 5(a) when the temperature below the 150 °C, then become single-vortex domain when the temperature increases to 151 °C. This is attributed to the compressive misfit strain tends to align the polarization dipoles along the same direction and thus results in the single-vortex domain structure formed^24^. The domain transformation is monodomain → single-vortex when the temperature increases from 165 to 675 °C. It is noted that the ECE of the PTO nanoparticle depended on the change of the polarization change. A large polarization change can be found during the domain structure transformation happens. Therefore, the Δ*T* increases linearly in region I mainly attributing to double-vortex → single-vortex domain transformation. And in region II shown in Fig. 4(b), Δ*T* changes as a fourth order curve attributing to the monodomain → single-vortex domain transformation.

## Conclusion

In this work, a double-vortex domain structure is obtained, and the ECE of PbTiO_3_ nanoparticle with double-vortex domain is studied by using phase field method. Giant adiabatic temperature change is obtained in the temperature range 0 ~ 675 °C, and the lowest and highest adiabatic temperature change are 7.2 K and 16.5 K, respectively. When the misfit strain exists between the ferroelectric material and substrate material, compress misfit strain can enhance the ECE of PbTiO_3_ nanoparticle with double-vortex domain, however, tensile misfit strain can reduce the ECE. This work reveals a way to obtain giant ECE of ferroelectric materials.

## Methods

In this work, phase field theory is employed to simulate the domain structure of ferroelectric materials. The polarization ***P*** = (*P*_1_, *P*_2_, *P*_3_) is chosen as the order parameter to describe the domain structure of PbTiO_3_ nanoparticle, which can be obtained by solving the time-dependent Ginzburg-Landau equation,

1 $$\frac{\partial {P}_{i}({\boldsymbol{r}},t)}{\partial t}=-L\frac{\delta F}{\delta {P}_{i}({\boldsymbol{r}},t)}\,\,\,(i=1,2,3)$$

where L is kinetic coefficient, *F* is the total free energy of PbTiO_3_ nanoparticle, ***r*** is spatial vector. The total free energy ***F*** of PbTiO_3_ nanoparticle can be written as^25^,

2 $$F={\int }_{V}[{f}_{LD}({P}_{i})+{f}_{G}({P}_{i,j})+{f}_{elas}({P}_{i},{\varepsilon }_{ij})+{f}_{elec}({P}_{i},{E}_{i}^{a})]dV$$

where *f*_*LD*_ is Landau-Devonshire energy density, *f*_*G*_ is gradient energy density, *f*_*elas*_ is elastic energy density, *f*_*elec*_ is electric energy density, and *V* denotes volume. The energy density *f*_*LD*_, *f*_*G*_, *f*_*elas*_, *f*_*elec*_ can be expressed as following equations, respectively^26,27^,

3 $$\begin{array}{c}{f}_{LD}({P}_{i})={\alpha }_{1}({P}_{1}^{2}+{P}_{2}^{2}+{P}_{3}^{2})+{\alpha }_{11}({P}_{1}^{4}+{P}_{2}^{4}+{P}_{3}^{4})\\ \,\,\,\,\,\,\,\,+\,{\alpha }_{12}({p}_{1}^{2}{P}_{2}^{2}+{P}_{2}^{2}{P}_{3}^{2}+{P}_{1}^{2}{P}_{3}^{2})+{\alpha }_{111}({P}_{1}^{6}+{P}_{2}^{6}+{P}_{3}^{6})\\ \,\,\,\,\,\,\,\,+\,{\alpha }_{112}[{P}_{1}^{4}({P}_{2}^{2}+{P}_{3}^{2})+{P}_{2}^{4}({P}_{1}^{2}+{P}_{3}^{2})+{P}_{3}^{4}({P}_{1}^{2}+{P}_{2}^{2})]\,\,\,\\ \,\,\,\,\,\,\,\,+\,{\alpha }_{123}{P}_{1}^{2}{P}_{2}^{2}{P}_{3}^{2}\end{array}$$

4 $$\begin{array}{c}{f}_{G}({P}_{i,j})=\frac{1}{2}{G}_{11}({P}_{1,1}^{2}+{P}_{2,2}^{2}+{P}_{3,3}^{2})+{G}_{12}({P}_{1,1}{P}_{2,2}+{P}_{1,1}{P}_{3,3}+{P}_{3,3})\\ \,\,\,\,\,\,\,\,+\,\frac{1}{2}{G}_{44}[{({P}_{1,2}+{P}_{2,1})}^{2}+{({P}_{2,3}+{P}_{3,2})}^{2}+{({P}_{1,3}+{P}_{3,1})}^{2}]\\ \,\,\,\,\,\,\,\,+\,\frac{1}{2}{G}_{44}^{^{\prime} }[{({P}_{1,2}-{P}_{2,1})}^{2}+{({P}_{2,3}-{P}_{3,2})}^{2}+({P}_{1,3}-{P}_{3,1})]\end{array}$$

5 $${f}_{{elas}}({P}_{i},{\varepsilon }_{ij})=\frac{1}{2}{c}_{ijkl}({\varepsilon }_{ij}-{\varepsilon }_{ij}^{0})({\varepsilon }_{kl}-{\varepsilon }_{kl}^{0})$$

6 $${f}_{elec}({P}_{i},{E}_{i}^{a})={f}_{ap}+{f}_{dep}$$

where *α*_1_, *α*_11_, *α*_12_, *α*_111_, *α*_112_, α_123_ are the dielectric stiffness in equation (3), and *α*_1_ = (*T*−*T*_0_)/2ε_0_ *C*_0_. *ε*_0_ is the dielectric constant of vacuum, *C*_0_ is the Curie constant, *T* and *T*_0_ denote the temperature and the Curie-Weiss temperature, respectively. *G*_11_, *G*_12_, *G*_44_ and ${G}_{44}^{\text{'}}$ are gradient energy coefficients in equation (4). *c*_*ijkl*_ are the elastic energy constants in equation (5), and *ε*_*ij*_ is the total strain and ${\varepsilon }_{ij}^{0}$ is the eigenstrain. *f*_*ap*_ and *f*_*dep*_ denote the additional electric energy density and depolarization energy density, respectively, in equation (6). And *f*_*ap*_ and *f*_*dep*_ can be written as ${f}_{ap}=-{E}_{i}^{a}{P}_{i}$ and ${f}_{dep}=-\frac{1}{2}\sum ({E}_{i}^{d}{P}_{i})$, where ${E}_{i}^{a}$ and ${E}_{i}^{d}$ represents the component of external electric field and depolarization field along *x*_i_ axe, respectively.

The adiabatic temperature change (Δ*T*) can be calculated by using the following equation^28^,

7 $${\rm{\Delta }}T=-\frac{T}{\rho {C}_{E}}{\int }_{{E}_{a}}^{{E}_{b}}{(\frac{\partial P}{\partial T})}_{E}dE$$

where Maxwell relation ${(\frac{\partial P}{\partial T})}_{\sigma ,E}={(\frac{\partial S}{\partial E})}_{\sigma ,T}$ is used. *ρ* is the density of material, *C*_*E*_ is the heat capacity. *E*_*a*_ and *E*_*b*_ denote initial and final electric field, respectively, *T* is the temperature which the unit is K, *S* is entropy and *σ* is stress.
